# Supplementary material for: Pressure, motion, and conformational entropy in molecular recognition by proteins
Source: Biophys Rep (N Y). 2022 Dec 28;3(1):100098. doi: 10.1016/j.bpr.2022.100098 (PMC9840116; doi:10.1016/j.bpr.2022.100098)
Supplement: Document S1. Figures S1 and S2 [file mmc1.pdf]

Biophysical Reports, Volume *3*

Supplemental information

Pressure, motion, and conformational entropy in molecular recognition by proteins

José A. Caro, Kathleen G. Valentine, Taylor R. Cole, and A. Joshua Wand

Supplementary Material

Pressure, motion and conformational entropy in molecular recognition by proteins

José A. Caro,[1] Kathleen G. Valentine[4], Taylor R. Cole[1] and A. Joshua Wand[1,2,3,4*]

Departments of Biochemistry & Biophysics,[1] Chemistry,[2] and Molecular & Cellular Medicine,[3] Texas A&M University, College Station, Texas 77843 USA, [4]Department of Biochemistry & Biophysics, Perelman School of Medicine, University of Pennsylvania, Philadelphia, Pennsylvania 19104 USA

*Correspondence: josh.wand@ag.tamu.edu

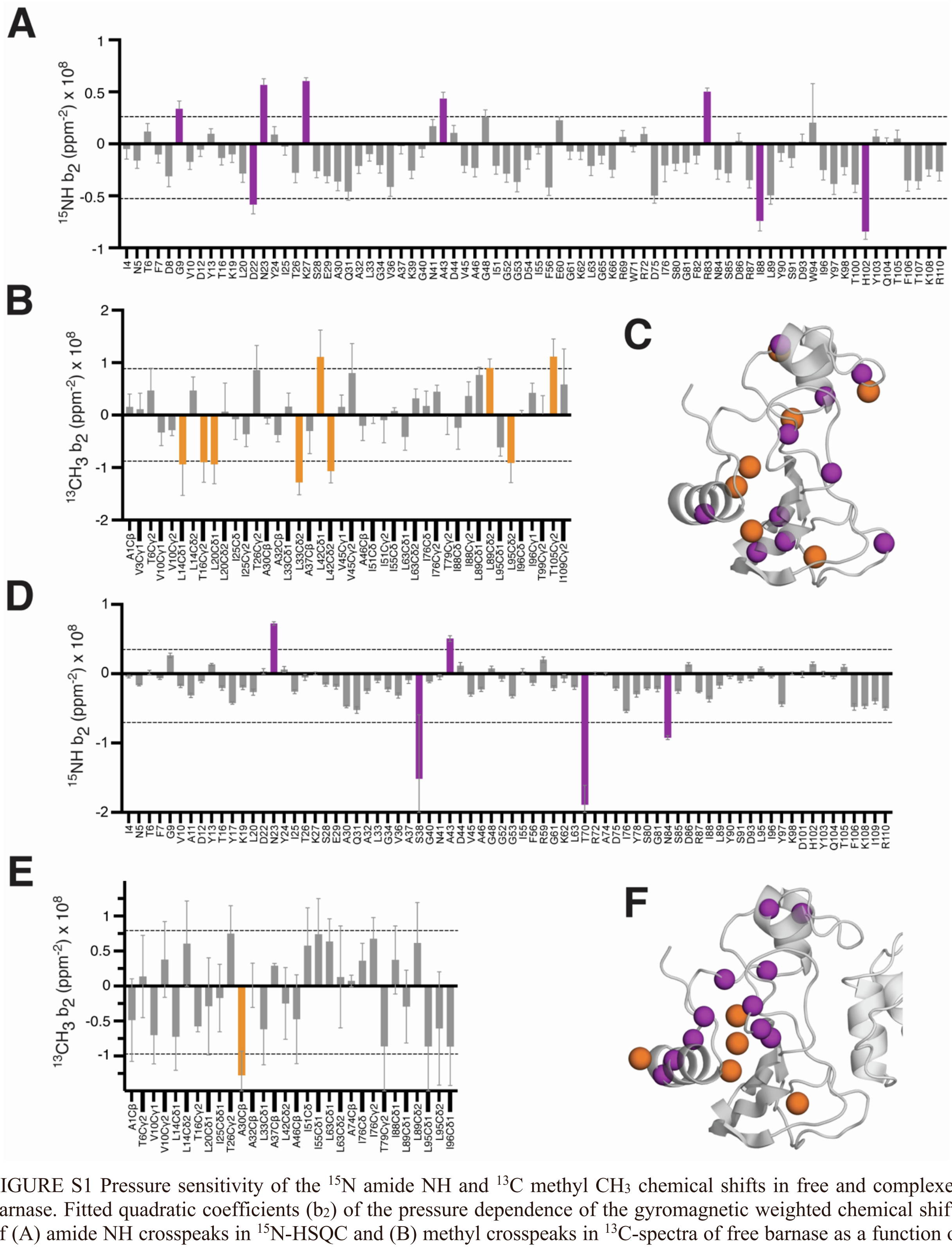

FIGURE S1 Pressure sensitivity of the ^{15}N amide NH and ^{13}C methyl CH_3 chemical shifts in free and complexed barnase. Fitted quadratic coefficients (b_2) of the pressure dependence of the gyromagnetic weighted chemical shifts of (A) amide NH crosspeaks in ^{15}N-HSQC and (B) methyl crosspeaks in ^{13}C-spectra of free barnase as a function of pressure. Error bars represent one standard deviation as derived from the fitting. Sites having quadratic coefficients greater than 1.5 standard deviations from the mean are highlighted on a ribbon representation of the ambient pressure structure (C). Amide and methyl groups are indicated as purple and orange spheres respectively. The pressure sensitivity of amide and methyl chemical shifts of barnase in complex with barstar is similarly shown (D, E, F).

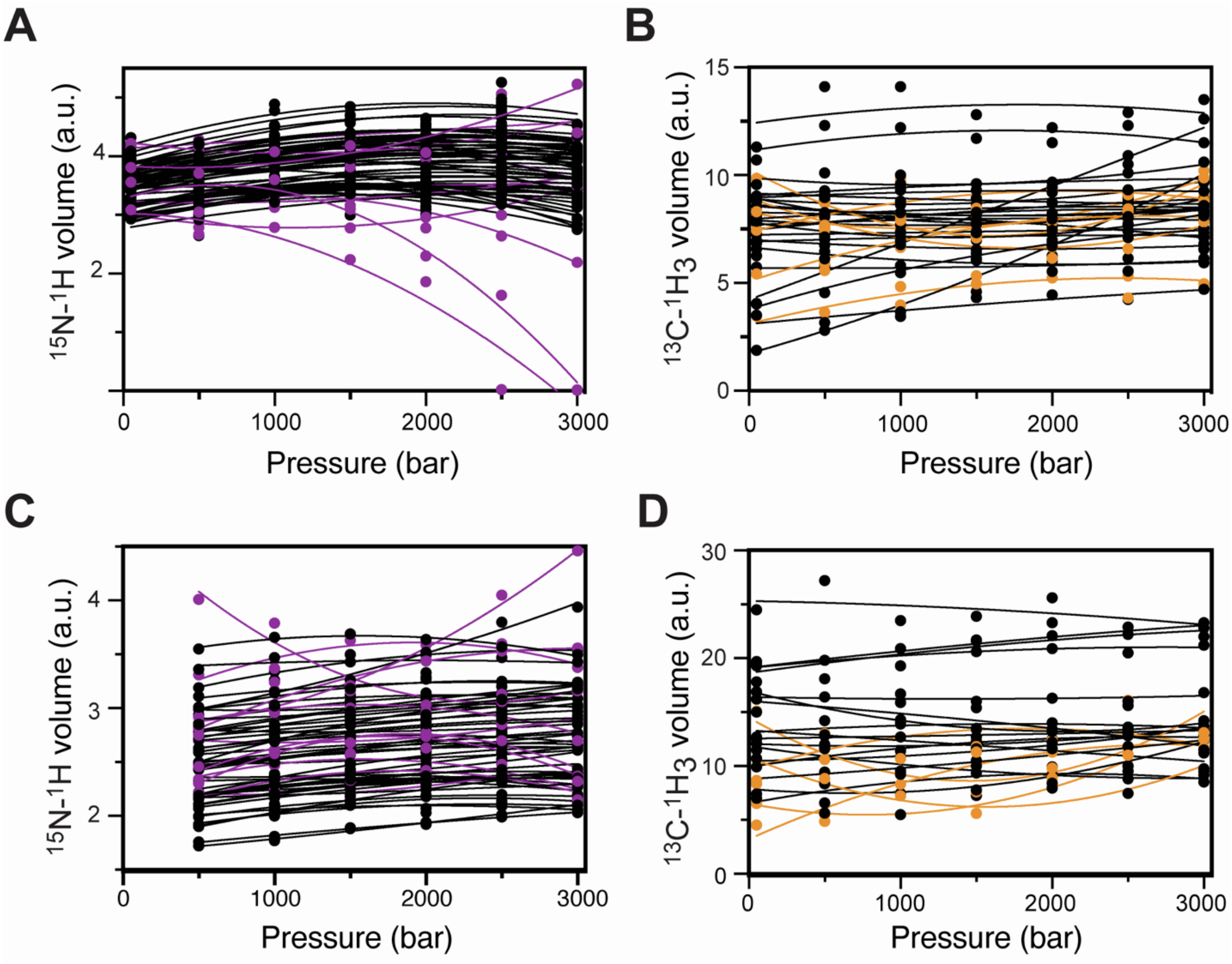

FIGURE S2 Pressure sensitivity of the fast internal motion of free and complexed barnase. Changes in volumes of (A) amide NH crosspeaks in ^{15}N-HSQC and (B) methyl crosspeaks in ^{13}C-spectra of free barnase and (C) amide NH crosspeaks in ^{15}N-HSQC and (D) methyl crosspeaks in ^{13}C-spectra of barnase in complex with barstar as a function of pressure. Lines fitted to a second order Taylor expansion are shown. Sites having b_2 coefficients greater than 1.5 standard deviations from the mean are indicated as purple and orange lines for amide and methyl crosspeaks, respectively. Fitted second order coefficients (b_2) are summarized in Fig. 3 of the main text.
