# Supplementary material for: Pressure, motion, and conformational entropy in molecular recognition by proteins
Source: Biophys Rep (N Y). 2022 Dec 28;3(1):100098. doi: 10.1016/j.bpr.2022.100098 (PMC9840116; doi:10.1016/j.bpr.2022.100098)
Supplement: Document S2. Article plus supporting material [file mmc2.pdf]

Biophysical Reports

REPORT

Pressure, motion, and conformational entropy in molecular recognition by proteins

José A. Caro,[1] Kathleen G. Valentine,[4] Taylor R. Cole,[1] and A. Joshua Wand[1,2,3,4,*]
[1]Department of Biochemistry & Biophysics, Texas A&M University, College Station, Texas; [2]Department of Molecular & Cellular Medicine, Texas A&M University, College Station, Texas; [3]Department of Chemistry, Texas A&M University, College Station, Texas; and [4]Department of Biochemistry & Biophysics, Perelman School of Medicine, University of Pennsylvania, Philadelphia, Pennsylvania

ABSTRACT The thermodynamics of molecular recognition by proteins is a central determinant of complex biochemistry. For over a half-century, detailed cryogenic structures have provided deep insight into the energetic contributions to ligand binding by proteins. More recently, a dynamical proxy based on NMR-relaxation methods has revealed an unexpected richness in the contributions of conformational entropy to the thermodynamics of ligand binding. Here, we report the pressure dependence of fast internal motion within the ribonuclease barnase and its complex with the protein barstar. In what we believe is a first example, we find that protein dynamics are conserved along the pressure-binding thermodynamic cycle. The femtomolar affinity of the barnase-barstar complex exists despite a penalty by $-T\Delta S_{conf}$ of $+11.7$ kJ/mol at ambient pressure. At high pressure, however, the overall change in side-chain dynamics is zero, and binding occurs with no conformational entropy penalty, suggesting an important role of conformational dynamics in the adaptation of protein function to extreme environments. Distinctive clustering of the pressure sensitivity is observed in response to both pressure and binding, indicating the presence of conformational heterogeneity involving less efficiently packed alternative conformation(s). The structural segregation of dynamics observed in barnase is striking and shows how changes in both the magnitude and the sign of regional contributions of conformational entropy to the thermodynamics of protein function are possible.

WHY IT MATTERS Conformational entropy is a potentially important contribution to the thermodynamics of molecular recognition by proteins. Fast internal motion has been developed as proxy for conformational entropy. NMR relaxation provides access to both backbone and side-chain dynamics on ns-ps timescales. Here, we determine the role of conformational entropy in the extremely high affinity barnase-barstar protein-protein interaction. We find that the contribution of conformational entropy to molecular complexation can be inverted by application of hydrostatic pressure. These results suggest that conformational dynamics, and the entropy that is represented, can provide a significant mechanism for adaptation to extreme environments.

INTRODUCTION

The change in the Gibbs free energy underlying molecular recognition and other complex protein functions such as allosteric regulation has, in principle, net contributions from both entropy and enthalpy. The latter is comprised of the internal energy and a pressure-volume work term. Detailed analysis of static low-temperature structural models has historically provided great insight into the internal energy and has promoted significant advances in understanding protein functions such as ligand binding through simulation and theory (1). Nevertheless, the origins of protein conformational entropy and its contribution to functions such as allostery remain much less well defined (2). Measurement of equilibrium fluctuations offers a powerful way to describe transitions between and occupancy of states that cannot be observed with classical methods of structural biology, and NMR relaxation has proven particularly useful in this regard (3–5). Over the past two decades, numerous studies of fast internal side-chain motion by NMR methods, particularly that of methyl-bearing amino acids, have revealed an unexpected complexity without distinguishing structural correlates (6).

Submitted October 10, 2022, and accepted for publication December 23, 2022.

*Correspondence: josh.wand@ag.tamu.edu

Editor: Paul Schanda.

https://doi.org/10.1016/j.bpr.2022.100098

© 2022 The Author(s).

This is an open access article under the CC BY license (http://creativecommons.org/licenses/by/4.0/).

Here, we take advantage of the fact that the Gibbs free-energy change associated with a change in state contains a pressure-volume work term. Volume changes represent the natural variable, and application of pressure can illuminate otherwise unobservable details of the thermodynamics of protein functions such as ligand binding and allostery. Protein molecules respond, both dynamically and structurally, to pressure in a complicated way. Pressure can compress proteins (7–9), remodel active sites (9), and facilitate excursions to higher-lying (10,11), locally unfolded (12–15), or globally unfolded states (16,17), thereby revealing various aspects of the ensemble nature of proteins. Here, we use high-pressure NMR (18) relaxation to probe fast internal motion of methyl-bearing side chains in the small enzyme barnase and use this motion as a proxy for changes in conformational entropy (ΔS_{conf}) (2,4).

MATERIALS AND METHODS

Sample preparation

pET-DUET expression plasmids containing the genes for barnase and barstar under the control of their own T7 promoter were obtained from GenScript Biotech Corporation (Piscataway, NJ, USA). An N-terminal 6xHis-tag followed by a Factor Xa cleavage site (MGSSHHHHHHSQAPIEGR) was added to barnase, while barstar remained untagged. Expression was carried out in BL21-(DE3) *E. coli* cells. Barstar expressed and purified with the N-terminal Met residue present. NMR-relaxation samples were prepared largely as described elsewhere (4). Deuterium (19) and ^{15}N relaxation (20) experiments of the free proteins were performed on a 1:2 mixture of uniformly ^{15}N-labeled protein and uniformly ^{13}C-labeled protein expressed in 60% D_2O media to generate the $^{13}CH_2D$ isotopomer. The complex was studied by combining ^{15}N-labeled protein (barnase or barstar) with $^{13}CH_2D$-labeled binding partner (barstar or barnase). Prochiral methyl assignment samples were expressed during growth on 10% $^{13}C_6$-glucose and 90% unlabeled glucose and uniform ^{15}N labeling (21).

The barnase-barstar complex was isolated by Ni-NTA affinity chromatography, and the complex dissociated with 6 M guanidine HCl (pH 7.9). Barstar was collected in the flow through (20 mL) and refolded by dilution into 1 L of water. Refolded barstar was further purified by a DEAE ion-exchange column that included a wash with 25 mM imidazole (pH 7.9) and 10 mM KCl and elution with 500 mM NaCl, being spin concentrated (3 kDa cutoff), and further purified by size-exclusion chromatography on Superdex SEC-75 equilibrated with 25 mM imidazole (pH 7.9) and 10 mM KCl. Barnase was eluted from the Ni-NTA column with 500 mM imidazole, spin concentrated (3 kDa cutoff), and buffer exchanged into 25 mM imidazole (pH 6.2), 10 mM KCl, and 5 mM $CaCl_2$. The His tag was cleaved with 4 μg factor Xa per mg of barnase added and mixed overnight at room temperature. The solution was passed through a 1 mL Ni-NTA column coupled to a SEC-75 column in 50 mM imidazole (pH 7.9) and 50 mM KCl, spin concentrated, and buffer exchanged to 25 mM imidazole (pH 6.2) and 10 mM KCl. NMR experiments were performed with samples prepared in 25 mM imidazole (pH 6.2), 10 mM KCl, 5% D_2O, and 0.02% NaN_3 (w/v). Samples were stable about 1 month for the free proteins and several months for the complex at 35°C.

NMR assignment and relaxation of free and bound barnase

All experiments were carried out at 35°C. Assignment experiments were done on a uniformly ^{13}C, ^{15}N-labeled sample, with only one protein in the complex labeled to reduce spectral crowding. Nonuniform sampling was used extensively for triple-resonance assignment spectra (22). Assignments were mapped to high pressure by collecting ^{13}C and ^{15}N heteronuclear single quantum coherence (HSQC) spectra every 500 bar. These experiments were collected either at 500 or 600 MHz. Carbon and nitrogen HSQC spectra were collected at 1, 50, 500, 1,000, 1,500, 2,000, 2,500, and 3,000 bar with a waiting period of 1 h between each. Spectra were collected during ramp up and ramp down of pressure, with no detectable difference observed between them. Chemical shift analysis utilized the gyromagnetic ratio weighted change in chemical shift of ^{1}H and ^{15}N (or ^{13}C) of bonded atoms resolved in two-dimensional correlation spectra.

Resonance peak heights and volumes were obtained using NMRFAM-SPARKY (23). Only fully resolved crosspeaks that fitted well to a Lorentzian lineshape to provide intensities were included. Peaks were integrated using the fitted Lorentzian function. The pressure dependence of gyromagnetic weighted chemical shifts were analyzed using a second-order Taylor expansion (24). The correlation between second- and first-order coefficients (24) was fitted with a linear regression with intercepts (6.5 $\pm$ 0.8) and (-16 $\pm$ 6) $\times$ 10^{-10} ppm bar^{-2} and slopes (-7.2 $\pm$ 0.2) and (-2.2 $\pm$ 0.2) $\times$ 10^{-5} bar^{-1} for amide proton and nitrogen, respectively, for free barnase and intercepts (-2.2 $\pm$ 0.4) and (10 $\pm$ 2) $\times$ 10^{-10} ppm bar^{-2} and slopes (-6.7 $\pm$ 0.1) and (-3.2 $\pm$ 0.1) $\times$ 10^{-5} bar^{-1} for amide proton and nitrogen, respectively, for complexed barnase.

Longitudinal and transverse relaxation was measured using HSQC spectra with nine interleaved delay points and three duplicates (delays 2, 5, and 8) for uncertainty estimation (each applied to itself and neighboring delay points 1–3, 4–6, and 7–9) (25). Maximum peak intensities and uncertainties were used to fit single-exponential decay curves with three parameters. ^{1}H-^{15}N nuclear Overhauser enhancement experiments were measured with a 5 s mixing time with and without irradiation of ^{1}H. Relaxation was measured at 500 and 600 MHz (^{1}H) for high-pressure experiments and 500, 600, and 750 MHz (^{1}H) for ambient-pressure experiments. Deuterium relaxation employed IzCzDz and IzCzDy experiments with "on-the-fly" IzCz compensation (26). High-pressure NMR relaxation experiments were carried out in a 3 kbar rated 5 mm o.d./3 mm i.d. ceramic NMR tube connected to a high-pressure Xtreme-60 pressure generator (Daedalus Innovations, Aston, PA, USA). The pressure medium was degassed water with a mineral oil interface with the sample. The effect of pressure on imidazole's pK_a is small (27). Relaxation measurements on the complex (24 kDa) were performed on uniformly ^{15}N-labeled barstar and uniformly ^{13}C-labeled barnase expressed during growth in 60% D_2O media.

The macromolecular rotational correlation model and Lipari-Szabo squared generalized order parameters of the amide N–H bond vectors (O^2_{NH}) were determined from ^{15}N relaxation experiments (28). Tumbling models for the complex were determined using data from ^{15}N-labeled barstar backbone relaxation measurements and were chosen according to the Akaike and Bayesian information criteria and F-tests to obtain probability values for each model (29). Simple model-free parameters were determined using a grid-search in a C++/AMP implementation of Relxn2A (4,30). The analysis used an N–H bond length of 1.02 Å (31), ignoring any influence of angular motion of the bonded H; a general ^{15}N tensor breadth of -170 ppm (32); a quadrupolar coupling

TABLE 1 Fast internal motion in free barnase and barnase in the barnase-barstar complex

Barnase[a,b]	P (bar)	$\langle O^2_{axis} \rangle$	$varO^2_{axis}$ [c]	n_{NH} [d]	$\langle O^2_{NH} \rangle$	$varO^2_{NH}$	τ_m(ns)
Free (all)[e]	1	0.653	0.046	79	0.784	0.0014	4.8[f]
Free (region I)[g]	1	0.721	0.031	–	–	–	–
Free (region II)[h]	1	0.621	0.030	–	–	–	–
Free (all)	3,000	0.647	0.054	77	0.798	0.0017	5.9[i]
Free (region I)	3,000	0.762	0.029	–	–	–	–
Free (region II)	3,000	0.568	0.039	–	–	–	–
Complex (all)	1	0.693	0.055	66	0.725	0.0017	8.9[f]
Complex (region I)	3,000	0.725	0.023	–	–	–	–
Complex (region II)	1	0.667	0.040	–	–	–	–
Complex (all)	3,000	0.649	0.051	53	0.851	0.0023	11.5[i]
Complex (region I)	3,000	0.725	0.023	–	–	–	–
Complex (region II)	3,000	0.589	0.048	–	–	–	–

[a]All data obtained at 308 K.

[b]Number of probes spectrally resolved in all four states are 33, 16, and 12 for all, region I, and region II, respectively.

[c]Variance is shown to indicate the variation of the dynamics within each region.

[d]Only those amide ^{15}N-^{1}H probes in barnase with small contributions from chemical exchange (Rex) were used to characterize tumbling and backbone motion.

[e]Includes all spectrally resolved methyl probes in barnase.

[f]Fully anisotropic tumbling model, effective τ_m reported.

[g]Region I includes the buried side chains of Val-10, Leu-14, Leu-42, and Ile-51 and the surface exposed side chains of Val-3, Ile-4, Thr-6, Leu-20, Thr-26, Ile-25, Leu-33, Val-45, and Thr-79.

[h]Region II includes the buried side chains of Leu-63, Ile-76, Ile-88, Leu-89, and Ile-96 and the surface exposed side chains of Leu-95, Thr-99, Thr-100, Thr-107, and Ile-109.

[i]Axially symmetric tumbling model, effective τ_m reported.

constant of 167 kHz (33); and a methyl rotation order parameter O^2_{rot} of 0.1107 assuming perfect tetrahedral geometry of the methyl carbon.

Voronoi volumes (34) are ideally suited to investigate the volume of buried atoms as "the sum of polyhedral volumes is exactly equal to the total space occupied by the points" (35). Voronoi volumes were determined with an in-house Cython program. Only structural models with a nominal resolution of <2.5 Å based on data obtained at cryogenic temperature for the protein with the same biological context as that of the NMR experiment (e.g., free versus complexed) were considered. Deposited structures were used without modification. When multiple copies of the protein were present in the asymmetric unit, the copy with the strongest electron density and highest-quality model was identified by visual inspection. The calculation finds the edges of the coordinates and defines a box with a 5 Å padding. A cubic grid is created with a step size of 0.01 Å. Each voxel is interrogated for the nearest heavy atom and assigned to it. Voxels within the van der Waals radius (36) of any atom were excluded. The sum of all voxels assigned to an atom represents the atom's Voronoi polyhedron and is used to calculate its volume. Side-chain volumes were summed starting at the Cβ and ending with the atoms with the same number of dihedral angles as the methyl group of interest. For example, Ile Cγ2 methyls will include the volume of both Cγ carbons and one Cβ atom, while Ile Cδ methyls will include one Cδ, both Cγ, and one Cβ atom. The surface was defined using a large probe (2.4 Å radius) to avoid fitting inside any internal cavities. The surface algorithm ignored ligands and waters, so binding pockets were considered open surfaces as well. The probe was moved through the grid to find all voxels where the probe fit without steric overlap, and protein atoms that came within 1 voxel of the probe were flagged as belonging to the surface. If any atom of a side chain (starting at the Cβ and ignoring backbone) contained a surface atom, the side chain was not considered buried. Alternative rotamers were analyzed by including all rotamers in the calculation, summing the volumes of all rotamers of a given side chain, and subtracting the van der Waals volume only once. All unoccupied void volume is obtained by this calculation including that which remains from perfect packing of spheres.

RESULTS

Barnase-barstar is one of the strongest protein-protein interactions known in biology. Its fM affinity derives from large enthalpic contributions at the interface (37). Individual entropic contributions sum to yield a negligible (~zero) contribution to the binding free energy (37). We sought here to learn the dynamical character of barnase in its primary functional states, i.e., free and bound to the inhibitor barstar, and to also learn how elevated hydrostatic pressure influences this interaction. We quantify the disorder of the methyl symmetry axis in terms of the Lipari-Szabo squared generalized order parameter (O^2_{axis}) (38) obtained using deuterium NMR-relaxation methods (19). The O^2_{axis} can range from a value of one, corresponding to complete rigidity within the molecular frame, to zero, which effectively corresponds to isotropic disorder. Importantly, only motion faster than the overall molecular reorientation of the protein contributes to O^2_{axis}.The four states of barnase examined have quite different average O^2_{axis} values, variances about those averages (Table 1), and distributions within the molecular structure (Fig. 1).

At ambient pressure, we find that complexation is accompanied by an overall rigidification of the

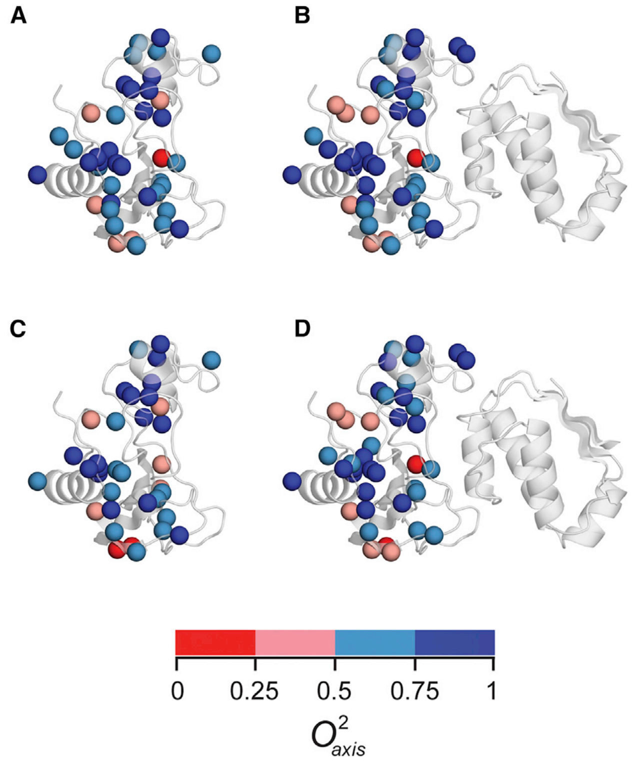

FIGURE 1 Methyl-bearing side chain motion in four states of barnase. Methyl dynamics of barnase mapped onto the structure as spheres colored according to O^2_{axis} value. (*A*) Free barnase. (*B*) Barnase bound to barstar. (*C*) Free barnase at 3 kbar. (*D*) Barnase bound to barstar at 3 kbar. At 35°C.

methyl-bearing side chains of barnase (Fig. 2; Table 1). Motion of side-chain torsions, both within a rotamer well and between rotamers, indirectly captures conformational entropy expressed on the timescales represented by the NMR-relaxation phenomena used here (2). Unfortunately, motion leading to interconversion of states slower than macromolecular tumbling are rendered invisible (38). For example, long-time molecular dynamics simulations of T4 lysozyme beyond the macromolecular tumbling regime suggest the presence of "excess" entropy (39), but it is not clear how higher-order couplings (40) influenced that estimate. Earlier, analysis of albeit somewhat shorter molecular dynamics simulations of seven proteins that removed the influence of coupled motions using the MIST algorithm (41) suggests that the vast majority of rotamer entropy is indeed expressed in order parameters obtained by solution NMR-relaxation approaches (42). Furthermore, Brüschweiler and co-workers utilized a clever strategy to sample timescales slower than macromolecular tumbling and found little influence on the methyl symmetry axis order parameters in ubiquitin (43). Nevertheless, to ameliorate this and other issues in the use of classical NMR-relaxation phenomena to characterize conformational entropy, the so-called NMR "entropy meter" was developed to provide an empirical calibration that avoids specific motional models and uses motion on the ps-ns timescale to capture changes in rotamer entropy (2). Long time (i.e., rare) fluctuations or coupling and shorter timescale-correlated motion is meant to be absorbed into the calibration of the entropy meter (2,4) and expressed in the limits of its determined precision. Accordingly, the dynamical proxy for conformational entropy (4) indicates that the overall rigidification of barnase upon complex formation corresponds to an unfavorable contribution ($\Delta S_{conf} < 0$) to the binding free energy corresponding to $+11.7 \pm 1.2$ kJ/mol at 300 K. Application of high hydrostatic pressure on free barnase yields an unexpected clustering of changes in motion (ΔO^2_{axis}) into two spatial regions, one that rigidifies with pressure and one that activates dynamically (Fig. 2; Table 1). Region I, which becomes more rigid with pressure, is defined by 21 methyl-bearing side chains that are largely localized to the N-terminal domain of the protein. Eight of these side chains are fully buried. Region II, which becomes more dynamic with applied pressure, is comprised of 17 methyl-bearing side chains in the C-terminal domain, and 11 of these are fully buried. Nine methyl-bearing side chains are outside of these regions. All methyl probes are 7 Å or more from the barnase-barstar interface, which is highly polar and extensively hydrated (44).

A thermodynamic cycle from free barnase was created with barnase either bound to its inhibitor barstar, subjected to high hydrostatic pressure (3 kbar), or both (Fig. 2). At 3 kbar, binding of barnase to barstar results in an opposite response from the N- and C-terminal groups of side chains. Motion in Region I is activated by elevated pressure, which is opposite to the response of free barnase. Region II rigidifies upon barnase binding barstar both at ambient and high pressure (Fig. 1; Table 1). Application of high pressure to the barnase-barstar complex leads to a general increase in the internal motion of barnase, with the largest change centered in region II. As might be expected, the pressure sensitivity of a side chain's motion is reduced as the O^2_{axis} approaches the rigid limit of one at ambient pressure. Of the four states of barnase examined, the complexed state at ambient pressure is the most rigid ($\langle O^2_{axis} \rangle = 0.693$) (Table 1).

The heterogeneous and regional response of side-chain dynamics to pressure is in stark contrast with other metrics examined. For example, fast backbone motions are generally suppressed in response to pressure and without apparent grouping to regions I and II (Table 1). The regional grouping in the dynamical character of the protein is also not apparent from the more usual tactic of characterizing the pressure dependence of NMR chemical shifts (Fig. S1).

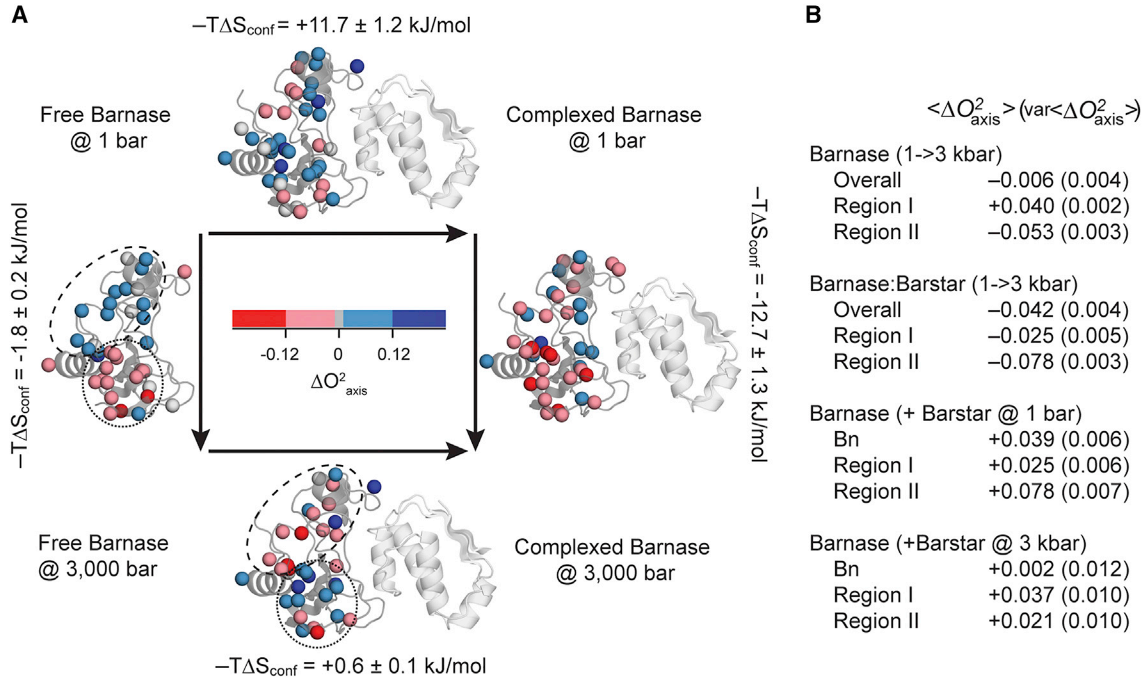

FIGURE 2 Dynamic response of barnase to high pressure and to binding barstar. (*A*) The locations of reporter methyl groups are indicated by spheres on the ribbon-diagram of barnase either free or in complex with barstar (PDB: 1B2X and 1B27) and color coded according to the ΔO^2_{axis}, where red and blue correspond to decreases and increases in ΔO^2_{axis} upon the indicated change of state, respectively. (*B*) Summary of the average changes of ΔO^2_{axis} values in the entire barnase molecule and within region I (*dashed line*) and region II (*dotted line*) of barnase under a pressure transition from 1 to 3 kbar and/or due to binding of barstar. Values in parentheses correspond to the variance of individual ΔO^2_{axis} values and not error. See the main text for details.

Nonlinear pressure-induced changes in chemical shifts in the free protein are heterogeneously distributed throughout the protein, while the complex shows very few chemical shifts with significant nonlinearity and provides little insight (Fig. S1).

In contrast, local conformational heterogeneity can be inferred from the response of crosspeak volumes to pressure in both free barnase and barnase in complex with barstar (Fig. 3). Crosspeak intensities in the free protein show a general increase with pressure, suggestive of a less heterogeneous conformational landscape (45), but also highlight three interfacial residues whose intensities collapse significantly over the span of 3 kbar. Spatial grouping of a nonlinear response to pressure is seen in the complex for 11 backbone and five methyl resonances, the large majority of which are located in region I (Fig. 3). As in the free state, most of these resonances show negative curvature and an initial gain in intensity with pressure, indicating that pressure leads to a more ordered backbone. Though locally heterogeneous in detail, the overall response of protein dynamics to changes along the thermodynamic cycle is, of course, conserved for both backbone and side chains (Fig. 2, *inset table*). Indeed, the indicated precision is remarkable.

DISCUSSION

Despite the development of high-pressure NMR sample tubes suitable for multidimensional heteronuclear NMR of proteins some time ago (46), there has been only one previous study of the pressure dependence of the fast ps-ns motions of methyl-bearing amino acid side chains (47). In that work, the motions of methyl-bearing side chains of human ubiquitin were found to be significantly perturbed by the application of hydrostatic pressures reaching to 2.5 kbar. As observed here for barnase, both free and in complex with its natural inhibitor barstar, ubiquitin side-chain motion showed a heterogeneous response with small volumes of "clustered" (i.e., spatially localized) perturbations of similar magnitude. However, the influence of applied pressure on methyl-bearing side-chain motion in free barnase and the barnase-barstar complex is more striking.

The localized response of motion to pressure is not easily explained by $\langle O^2_{axis}\rangle$ values at ambient pressure (region I: $\langle O^2_{axis}\rangle$ (*var* $\langle O^2_{axis}\rangle$) $= 0.721$ (0.031), n $= 16$; region II: $\langle O^2_{axis}\rangle$ (*var* $\langle O^2_{axis}\rangle$) $= 0.621$ (0.030), n $= 12$) (Fig. 1). Elevation to 3 kbar results in $\langle \Delta O^2_{axis}\rangle$ (*var* $\langle \Delta O^2_{axis}\rangle$) of $+0.040$ (0.002) and -0.053 (0.003) for Regions I and II, respectively, and suggests the

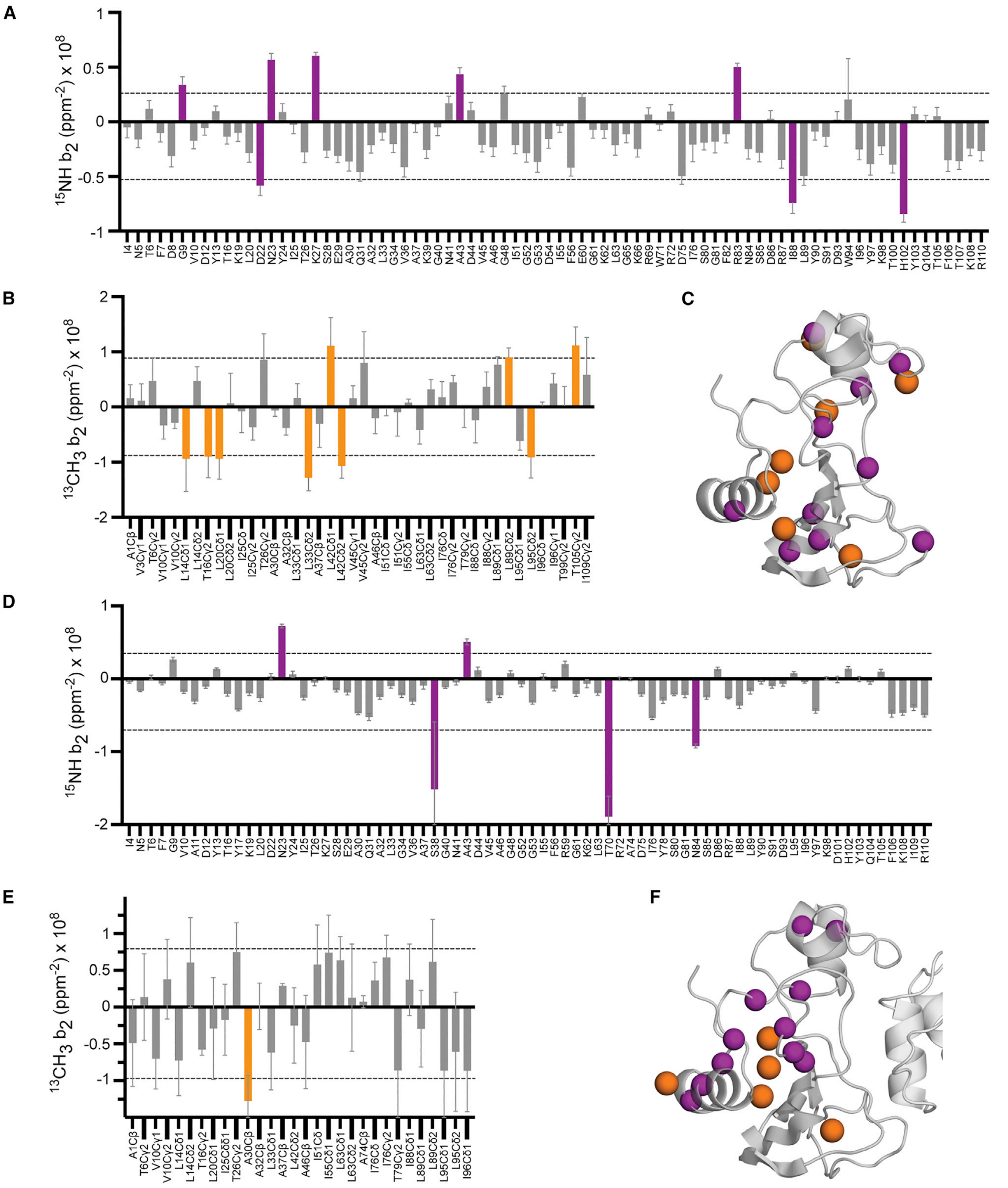

FIGURE 3 Pressure sensitivity of amide and methyl crosspeak intensities of free and complexed barnase. Fitted quadratic coefficients (b_2) of the pressure dependence of the volumes of (*A*) amide NH crosspeaks in ^{15}N HSQC and (*B*) methyl crosspeaks in ^{13}C spectra of free barnase as a function of pressure. Error bars represent one standard deviation as derived from the fitting. Sites b_2 values greater than 1.5 standard deviations from the mean are highlighted on ribbon representation of the ambient pressure structure (*C*). Amide and methyl groups are indicated as purple and orange spheres, respectively. The pressure sensitivity of amide and methyl crosspeak intensities for barnase in complex with barstar is similarly shown (*D*–*F*). Individually fitted crosspeak volumes versus pressure are shown in Fig. S2.

presence of some distinguishing property that results in their segregation. The effect of pressure is fundamentally related to changes in system volume. To examine potential contributions to the pressure sensitivity by the protein itself, we carried out a fine-grained volumetric analysis of the crystal structure (see materials and methods). Focusing on methyl-bearing side chains without solvent accessible surface area, we find that region I side chains in the ambient-pressure structure, on average, have ~35 Å^3 more unoccupied volume surrounding the side chain than those of region II (111 $\pm$ 23 and 78 $\pm$ 33 Å^3, respectively; $p < 0.022$). Compression of voids explains the rigidification by pressure observed in region I. In contrast, a more densely packed region II may not be able to further compress and will respond to pressure through other mechanisms that decrease the system volume, such as local structural transitions or changes in hydration. These initial observations have prompted a broader examination of experimentally determined methyl symmetry axis order parameters to investigate the influence of surrounding void volume more generally. These results will be presented elsewhere.

The barnase-barstar complex has perhaps the highest affinity known for a noncovalent heterodimer with a dissociation constant in the low femtomolar range (37). High affinity binding selectively alters the response of barnase to pressure. Region I rigidifies as free barnase is compressed, indicating that there is conformational heterogeneity involving less efficiently packed alternative conformation(s). Interestingly, this region corresponds to a putative late-folding intermediate (48). In distinct contrast, pressure favors increased disorder on the subnanosecond timescale in region II. As noted above, only very localized spatial clustering of the response of fast motion to pressure was observed in ubiquitin (47), but the extent of structural segregation in barnase is more pronounced and involves larger volumes of protein. The femtomolar affinity of the barnase-barstar complex exists despite a determined $-T\Delta S_{conf}$ penalty of +11.7 kJ/mol. But at high pressure, the overall change in side-chain dynamics is zero, and binding occurs with no conformational entropy penalty. This observed response of side chains to pressure is consistent with an important role of conformational entropy, reflected by changes in fast side-chain motion (4), in the adaptation of protein function to extreme environments (41). Furthermore, these results make clear that changes in both the magnitude and the sign of regional contributions of conformational entropy to the thermodynamics of protein function are possible.

DATA AVAILABILITY

The barnase relaxation data reported here has been deposited to the BMRB under BMRB: 50791 and 50792.

SUPPORTING MATERIAL

Supporting material can be found online at https://doi.org/10.1016/j.bpr.2022.100098.

AUTHOR CONTRIBUTIONS

A.J.W. and J.A.C. conceived and designed the project. K.G.V. and J.A.C. carried out the relaxation experiments and primary data analysis. J.A.C., K.G.V., T.R.C., and A.J.W. analyzed the results. A.J.W. and J.A.C. wrote the manuscript.

ACKNOWLEDGMENTS

This work was supported by the Mathers Foundation (MF-1809-00155), the National Institutes of Health (GM102447 and GM100910), an Extreme Biophysics Research Coordination Network grant MCB1817845 from the National Science Foundation, and Texas A&M University.

DECLARATION OF INTERESTS

A.J.W. is a founding member of Daedalus Innovations, LLC (Aston, PA, USA), a manufacturer of high-pressure NMR apparatus.

REFERENCES

1. Zhou, H. X., and M. K. Gilson. 2009. Theory of free energy and entropy in noncovalent binding. *Chem. Rev.* 109:4092–4107.
2. Wand, A. J., and K. A. Sharp. 2018. Measuring entropy in molecular recognition by proteins. *Annu. Rev. Biophys.* 47:41–61.
3. Mittermaier, A., and L. E. Kay. 2006. Review - New tools provide new insights in NMR studies of protein dynamics. *Science.* 312:224–228.
4. Caro, J. A., K. W. Harpole, ..., A. J. Wand. 2017. Entropy in molecular recognition by proteins. *Proc. Natl. Acad. Sci. USA.* 114:6563–6568.
5. Sekhar, A., and L. E. Kay. 2019. An NMR view of protein dynamics in health and disease. *Annu. Rev. Biophys.* 48:297–319.
6. Igumenova, T. I., K. K. Frederick, and A. J. Wand. 2006. Characterization of the fast dynamics of protein amino acid side chains using NMR relaxation in solution. *Chem. Rev.* 106:1672–1699.
7. Kundrot, C. E., and F. M. Richards. 1987. Crystal-structure of hen egg-white lysozyme at a hydrostatic-pressure of 1000 atmospheres. *J. Mol. Biol.* 193:157–170.
8. Collins, M. D., M. L. Quillin, ..., S. M. Gruner. 2007. Structural rigidity of a large cavity-containing protein revealed by high-pressure crystallography. *J. Mol. Biol.* 367:752–763.
9. Yamada, H., T. Nagae, and N. Watanabe. 2015. High-pressure protein crystallography of hen egg-white lysozyme. *Acta Crystallogr. D Biol. Crystallogr.* 71:742–753.
10. Bezsonova, I., D. M. Korzhnev, ..., L. E. Kay. 2006. Hydration and packing along the folding pathway of SH3 domains by pressure-dependent NMR. *Biochemistry.* 45:4711–4719.

11. Korzhnev, D. M., I. Bezsonova, ..., L. E. Kay. 2006. Probing the transition state ensemble of a protein folding reaction by pressure-dependent NMR relaxation dispersion. *J. Am. Chem. Soc.* 128:5262–5269.

12. Nucci, N. V., B. Fuglestad, ..., A. J. Wand. 2014. Role of cavities and hydration in the pressure unfolding of T-4 lysozyme. *Proc. Natl. Acad. Sci. USA.* 111:13846–13851.

13. Fuglestad, B., M. A. Stetz, ..., A. J. Wand. 2017. Solution NMR investigation of the response of the lactose repressor core domain dimer to hydrostatic pressure. *Biophys. Chem.* 231:39–44.

14. Roche, J., J. A. Caro, ..., C. A. Royer. 2012. Cavities determine the pressure unfolding of proteins. *Proc. Natl. Acad. Sci. USA.* 109:6945–6950.

15. Fuentes, E. J., and A. J. Wand. 1998. Local stability and dynamics of apocytochrome b(562) examined by the dependence of hydrogen exchange on hydrostatic pressure. *Biochemistry.* 37:9877–9883.

16. Hawley, S. A. 1971. Reversible pressure-temperature denaturation of chymotrypsinogen. *Biochemistry.* 10:2436–2442.

17. Lesch, H., C. Hecht, and J. Friedrich. 2004. Protein phase diagrams: The physics behind their elliptic shape. *J. Chem. Phys.* 121:12671–12675.

18. Caro, J. A., and A. J. Wand. 2018. Practical aspects of high-pressure NMR spectroscopy and its applications in protein biophysics and structural biology. *Methods.* 148:67–80.

19. Muhandiram, D. R., T. Yamazaki, ..., L. E. Kay. 1995. Measurement of 2H T1 and T1rho relaxation times in uniformly 13C-labeled and fractionally 2H-labeled proteins in solution. *J. Am. Chem. Soc.* 117:11536–11544.

20. Farrow, N. A., R. Muhandiram, ..., L. E. Kay. 1994. Backbone dynamics of a free and a phosphopeptide-complexed Src homology-2 domain studied by N-15 NMR relaxation. *Biochemistry.* 33:5984–6003.

21. Neri, D., T. Szyperski, ..., K. Wüthrich. 1989. Stereospecific nuclear magnetic-resonance assignments of the methyl-gropus of valine and leucine in the DNA-binding domain of the 434-repressor by biosynthetically directional fractional C13 labeling. *Biochemistry.* 28:7510–7516.

22. Robson, S., H. Arthanari, ..., G. Wagner. 2019. Nonuniform sampling for NMR spectroscopy. *Methods Enzymol.* 614:263–291.

23. Lee, W., M. Tonelli, and J. L. Markley. 2015. NMRFAM-SPARKY: enhanced software for biomolecular NMR spectroscopy. *Bioinformatics.* 31:1325–1327.

24. Beck Erlach, M., J. Koehler, ..., H. R. Kalbitzer. 2014. Relationship between nonlinear pressure-induced chemical shift changes and thermodynamic parameters. *J. Phys. Chem. B.* 118:5681–5690.

25. Stetz, M. A., J. A. Caro, ..., A. J. Wand. 2019. Characterization of internal protein dynamics and conformational entropy by NMR relaxation. *Methods Enzymol.* 615:237–284.

26. Millet, O., D. R. Muhandiram, ..., L. E. Kay. 2002. Deuterium spin probes of side-chain dynamics in proteins. 1. Measurement of five relaxation rates per deuteron in C-13-labeled and fractionally H-2-enriched proteins in solution. *J. Am. Chem. Soc.* 124:6439–6448.

27. Quinlan, R. J., and G. D. Reinhart. 2005. Baroresistant buffer mixtures for biochemical analyses. *Anal. Biochem.* 341:69–76.

28. Tjandra, N., S. E. Feller, ..., A. Bax. 1995. Rotational diffusion anisotropy of human ubiquitin from ^{15}N NMR relaxation. *J. Am. Chem. Soc.* 117:12562–12566.

29. Moorman, V. R., K. G. Valentine, ..., A. J. Wand. 2014. Dynamic and thermodynamic response of the Ras protein Cdc42Hs upon association with the effector domain of PAK3. *J. Mol. Biol.* 426:3520–3538.

30. Dellwo, M. J., and A. J. Wand. 1989. Model-independent and model-dependent analysis of the global and internal dynamics of cyclosporine-A. *J. Am. Chem. Soc.* 111:4571–4578.

31. Yao, L., B. Vögeli, ..., A. Bax. 2008. NMR determination of amide N-H equilibrium bond length from concerted dipolar coupling measurements. *J. Am. Chem. Soc.* 130:16518–16520.

32. Lee, A. L., and A. J. Wand. 1999. Assessing potential bias in the determination of rotational correlation times of proteins by NMR relaxation. *J. Biomol. NMR.* 13:101–112.

33. Mittermaier, A., and L. E. Kay. 1999. Measurement of methyl H-2 quadrupolar couplings in oriented proteins. How uniform is the quadrupolar coupling constant? *J. Am. Chem. Soc.* 121:10608–10613.

34. Voronoi, G. 1908. New applications of continuous parameters to the theory of quadratic forms. - First memoir - Some properties of perfect positive quadratic forms. *J. für die Reine Angewandte Math. (Crelle's J.).* 133:97–178.

35. Richards, F. M. 1977. Areas, volumes, packing, and protein structure. *Annu. Rev. Biophys. Bioeng.* 6:151–176.

36. Bondi, A. 1964. van der Waals volumes and radii. *J. Phys. Chem. A.* 68:441–451.

37. Schreiber, G., and A. R. Fersht. 1995. Energetics of protein-protein interactions - Analysis of the barnase-barstar interface by single mutations and double mutant cycles. *J. Mol. Biol.* 248:478–486.

38. Lipari, G., and A. Szabo. 1982. Model-free approach to the interpretation of nuclear magnetic resonance relaxation in macromolecules. 1. Theory and range of validity. *J. Am. Chem. Soc.* 104:4546–4559.

39. Hoffmann, F., F. A. A. Mulder, and L. V. Schäfer. 2022. How much entropy is contained in NMR relaxation parameters? *J. Phys. Chem. B.* 126:54–68.

40. King, B. M., and B. Tidor. 2009. MIST: Maximum information spanning trees for dimension reduction of biological data sets. *Bioinformatics.* 25:1165–1172.

41. Ando, N., B. Barquera, ..., M. B. Watkins. 2021. The molecular basis for life in extreme environments. *Annu. Rev. Biophys.* 50:343–372.

42. Kasinath, V., K. A. Sharp, and A. J. Wand. 2013. Microscopic insights into the NMR relaxation-based protein conformational entropy meter. *J. Am. Chem. Soc.* 135:15092–15100.

43. Xiang, X., A. L. Hansen, ..., R. Brüschweiler. 2021. Observation of sub-microsecond protein methyl-side chain dynamics by nanoparticle-assisted NMR spin relaxation. *J. Am. Chem. Soc.* 143:13593–13604.

44. Buckle, A. M., G. Schreiber, and A. R. Fersht. 1994. Protein-protein recognition - Crystal structural analysis of barnase barstar complex at 2.0 Å resolution. *Biochemistry.* 33:8878–8889.

45. Maeno, A., D. Sindhikara, ..., R. Kitahara. 2015. Cavity as a source of conformational fluctuation and high-energy state: High-pressure NMR study of a cavity-enlarged mutant of T4 lysozyme. *Biophys. J.* 108:133–145.

46. Urbauer, J. L., M. R. Ehrhardt, ..., A. J. Wand. 1996. High-resolution triple-resonance NMR spectroscopy of a novel calmodulin peptide complex at kilobar pressures. *J. Am. Chem. Soc.* 118:11329–11330.

47. Fu, Y., V. Kasinath, ..., A. J. Wand. 2012. Coupled motion in proteins revealed by pressure perturbation. *J. Am. Chem. Soc.* 134:8543–8550.

48. Bycroft, M., A. Matouschek, ..., A. R. Fersht. 1990. Detection and characterization of a folding intermediate in barnase by NMR. *Nature.* 346:488–490.

Biophysical Reports, Volume *3*

Supplemental information

Pressure, motion, and conformational entropy in molecular recognition by proteins

José A. Caro, Kathleen G. Valentine, Taylor R. Cole, and A. Joshua Wand

Supplementary Material

Pressure, motion and conformational entropy in molecular recognition by proteins

José A. Caro,[1] Kathleen G. Valentine[4], Taylor R. Cole[1] and A. Joshua Wand[1,2,3,4*]

Departments of Biochemistry & Biophysics,[1] Chemistry,[2] and Molecular & Cellular Medicine,[3] Texas A&M University, College Station, Texas 77843 USA, [4]Department of Biochemistry & Biophysics, Perelman School of Medicine, University of Pennsylvania, Philadelphia, Pennsylvania 19104 USA

*Correspondence: josh.wand@ag.tamu.edu

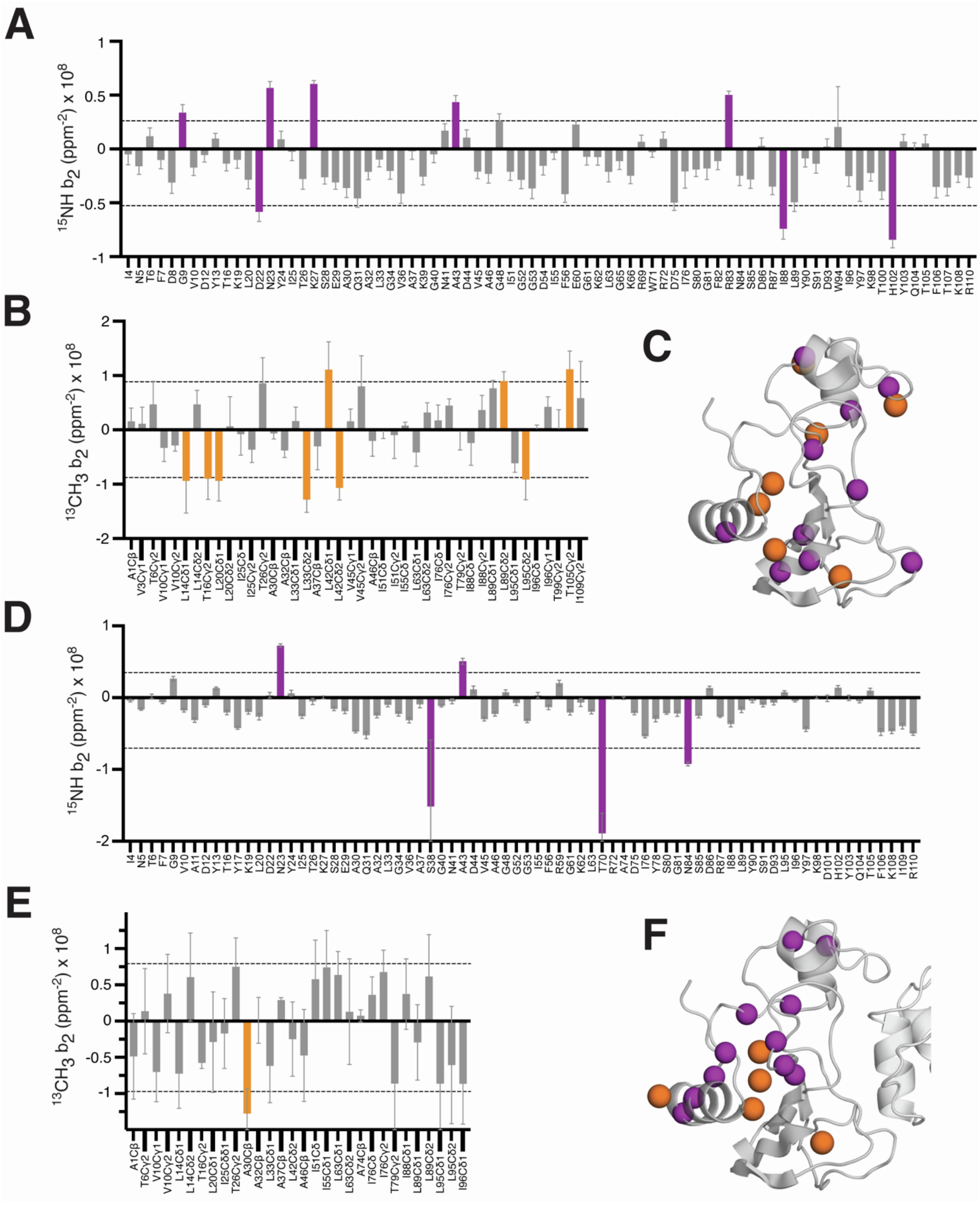

FIGURE S1 Pressure sensitivity of the ^{15}N amide NH and ^{13}C methyl CH_3 chemical shifts in free and complexed barnase. Fitted quadratic coefficients (b_2) of the pressure dependence of the gyromagnetic weighted chemical shifts of (A) amide NH crosspeaks in ^{15}N-HSQC and (B) methyl crosspeaks in ^{13}C-spectra of free barnase as a function of pressure. Error bars represent one standard deviation as derived from the fitting. Sites having quadratic coefficients greater than 1.5 standard deviations from the mean are highlighted on a ribbon representation of the ambient pressure structure (C). Amide and methyl groups are indicated as purple and orange spheres respectively. The pressure sensitivity of amide and methyl chemical shifts of barnase in complex with barstar is similarly shown (D, E, F).

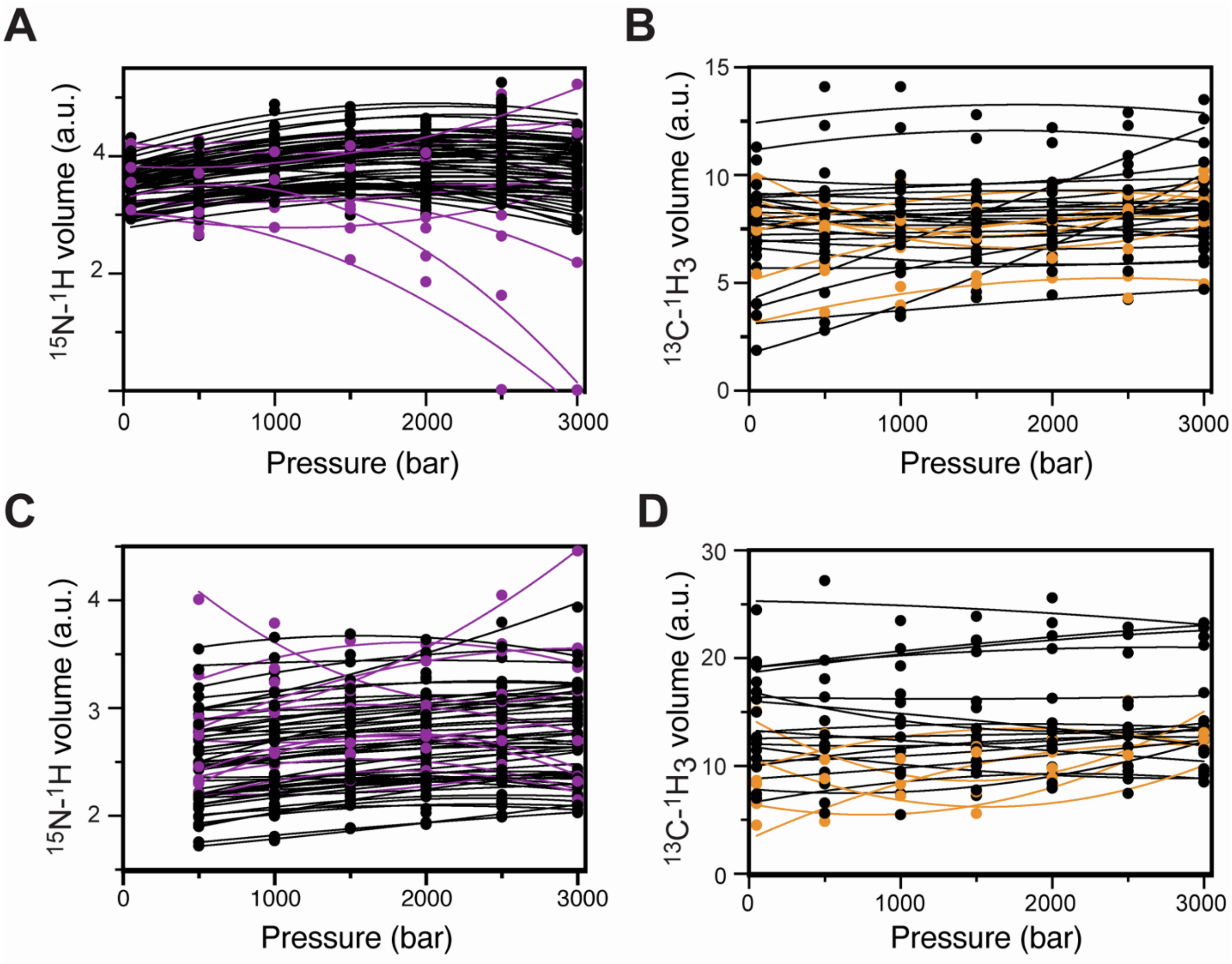

FIGURE S2 Pressure sensitivity of the fast internal motion of free and complexed barnase. Changes in volumes of (A) amide NH crosspeaks in ^{15}N-HSQC and (B) methyl crosspeaks in ^{13}C-spectra of free barnase and (C) amide NH crosspeaks in ^{15}N-HSQC and (D) methyl crosspeaks in ^{13}C-spectra of barnase in complex with barstar as a function of pressure. Lines fitted to a second order Taylor expansion are shown. Sites having b_2 coefficients greater than 1.5 standard deviations from the mean are indicated as purple and orange lines for amide and methyl crosspeaks, respectively. Fitted second order coefficients (b_2) are summarized in Fig. 3 of the main text.
